# Consequences of being overweight or obese during pregnancy on diabetes in the offspring: a record linkage study in Aberdeen, Scotland

**DOI:** 10.1007/s00125-019-4891-4

**Published:** 2019-06-19

**Authors:** Marius Lahti-Pulkkinen, Sohinee Bhattacharya, Sarah H. Wild, Robert S. Lindsay, Katri Räikkönen, Jane E. Norman, Siladitya Bhattacharya, Rebecca M. Reynolds

**Affiliations:** 1 British Heart Foundation Centre for Cardiovascular Science, Queen’s Medical Research Institute, University of Edinburgh, 47 Little France Crescent, Edinburgh, EH14 6TJ UK; 2 Tommy’s Centre for Maternal and Fetal Health, Medical Research Council Centre for Reproductive Health, Queen’s Medical Research Institute, University of Edinburgh, Edinburgh, UK; 3 Department of Psychology and Logopedics, Faculty of Medicine, University of Helsinki, Helsinki, Finland; 4 Institute of Applied Health Sciences, University of Aberdeen, Aberdeen, UK; 5 Usher Institute of Population Health Sciences and Informatics, University of Edinburgh, Edinburgh, UK; 6 Institute of Cardiovascular and Medical Sciences, University of Glasgow, Glasgow, UK

**Keywords:** Diabetes, Obesity, Offspring, Pregnancy

## Abstract

**Aims/hypothesis:**

Maternal obesity in pregnancy is associated with cardiovascular disease and mortality rate in the offspring. We aimed to determine whether maternal obesity is also associated with increased incidence of type 2 and type 1 diabetes in the offspring, independently of maternal diabetes as a candidate mechanistic pathway.

**Methods:**

Birth records of 118,201 children from 1950 to 2011 in the Aberdeen Maternity and Neonatal Databank were linked to Scottish Care Information–Diabetes, the national register for diagnosed diabetes in Scotland, to identify incident and prevalent type 1 and type 2 diabetes up to 1 January 2012. Maternal BMI was calculated from height and weight measured at the first antenatal visit. The effect of maternal obesity on offspring outcomes was tested using time-to-event analysis with Cox proportional hazards regression to compare outcomes in offspring of mothers in underweight, overweight or obese categories of BMI, compared with offspring of women with normal BMI.

**Results:**

Offspring of obese (BMI ≥30 kg/m^2^) and overweight (BMI 25–29.9 kg/m^2^) mothers had an increased hazard of type 2 diabetes compared with mothers with normal BMI, after adjustment for gestation when weight was measured, maternal history of diabetes before pregnancy, maternal history of hypertension, age at delivery, parity, socioeconomic status, and sex of the offspring: HR 3.48 (95% CI 2.33, 5.06) and HR 1.39 (1.06, 1.83), respectively.

**Conclusions/interpretation:**

Maternal obesity is associated with increased incidence of type 2 diabetes in the offspring. Evidence-based strategies that reduce obesity among women of reproductive age and that might reduce the incidence of diabetes in their offspring are urgently required.

**Electronic supplementary material:**

The online version of this article (10.1007/s00125-019-4891-4) contains peer-reviewed but unedited supplementary material, which is available to authorised users.

## Introduction

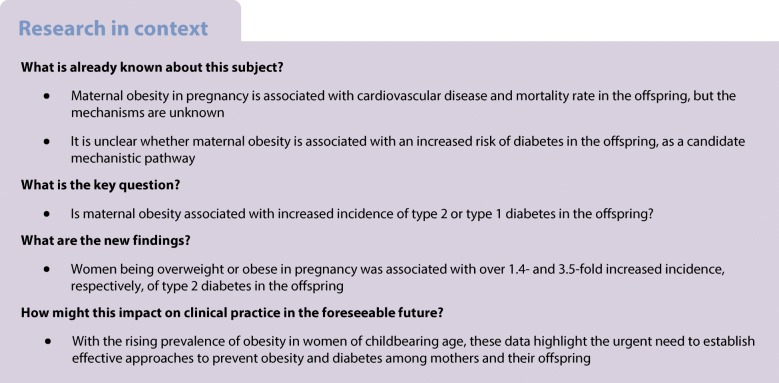



The short-term pregnancy complications of maternal obesity, including gestational diabetes, pre-eclampsia, need for Caesarean section and delivery of large-for-gestational age infants, are well recognised [1]. There is now increasing awareness that infants born to obese mothers have longer-term health problems, including increased risk of premature cardiovascular disease and premature mortality [2, 3]. As the prevalence of being overweight or obese among women of childbearing age is increasing worldwide [4], there is an urgent need to determine any long-term adverse effects for the offspring.

One key mechanistic pathway that may explain the link between maternal obesity and offspring cardiovascular disease [2, 3] is through increased risk of offspring type 2 diabetes. In support of this, higher maternal BMI was associated with cardiovascular risk factors, including increased fasting insulin (a surrogate marker of insulin resistance), among offspring aged 32 years in the Jerusalem Perinatal Family Follow-up Study [5]. In longer follow-up of older individuals born between 1934 and 1944 in the Helsinki Birth Cohort Study, mothers being overweight was associated with increased risk of offspring diabetes, particularly among female offspring [3]. In this study, however, the diagnosis of diabetes was based on the use of medication and so did not include individuals treated with diet alone and further could not distinguish between diagnoses of type 2 and type 1 diabetes. High maternal BMI has been identified as a risk factor for childhood-onset offspring type 1 diabetes in some studies [6–8] but not in others [9, 10].

One small case−control study reported a link between maternal retrospective recall of obesity in pregnancy and offspring type 2 diabetes in adolescence [11]. Another small study, conducted in South India, including follow-up of 204 individuals, found that increased maternal weight measured during pregnancy predicted higher risk of type 2 diabetes in adult offspring [12], although in this study the mean maternal weight was 47 kg. Yet, to our knowledge, no studies have specifically examined whether maternal obesity measured during pregnancy increases the risk of clinician-diagnosed type 2 diabetes in the offspring. Further, although 50% of type 1 diabetes develops after age 16 years [13], we are not aware of any studies examining whether the previously reported association between maternal obesity and increased risk of offspring childhood-onset type 1 diabetes [6–8] extends to type 1 diabetes diagnosed in adulthood.

We hypothesised that maternal obesity would increase the incidence of subsequent development of both type 2 and type 1 diabetes in the offspring. To test this hypothesis we examined the risk of developing a clinically confirmed diagnosis of type 2 or type 1 diabetes in 118,201 individuals aged 1–62 years whose mothers’ BMI was measured in pregnancy. We used a large pregnancy database, the Aberdeen Maternity and Neonatal Databank (AMND), linked to the Scottish Care Information–Diabetes (SCI-Diabetes) database, a national dataset for people with diagnosed diabetes in Scotland.

## Methods

**Fig. 1:**
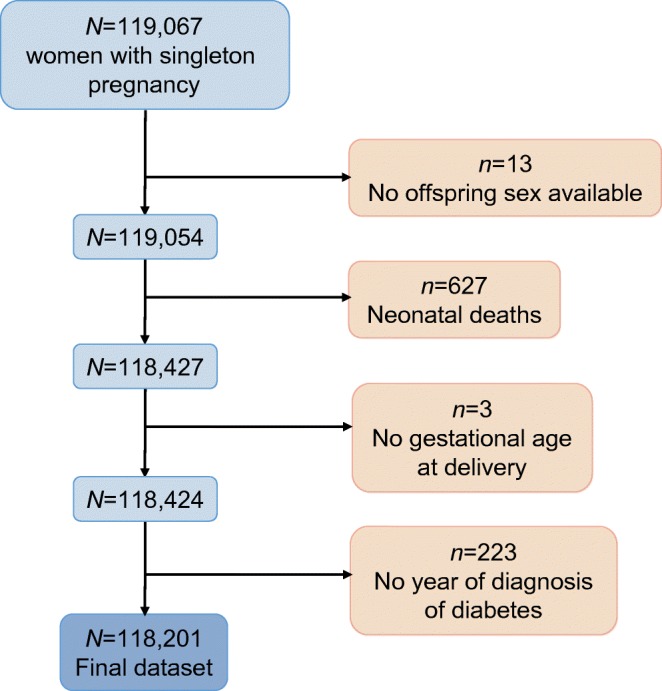
Flowchart. A linked dataset was created from 118,201 mothers and their offspring from the AMND databank linked to the SCI-Diabetes database, after excluding neonatal deaths and missing data for offspring sex, gestational age at delivery and year of diabetes diagnosis

In this cohort analysis, the exposure was maternal obesity during pregnancy and the outcomes in the offspring were: (1) any diabetes; (2) type 1 diabetes; (3) type 2 diabetes. We created a linked dataset including data from 118,201 mothers and their offspring from the AMND databank linked to the SCI-Diabetes database after excluding neonatal deaths and for missing data for offspring sex, gestational age at delivery and year of diabetes diagnosis (Fig. 1). The AMND is a unique databank linking all the obstetric and fertility-related events occurring in women from a defined geographical area with a relatively stable population, between 1950 and the present day [14]. Completeness of the database is checked annually against the number of deliveries recorded in the National Health Service records office, and validity of data entry is verified by numerous consistency checks. All women who delivered a live singleton infant at term (≥37 weeks’ gestation) between 1950 and 2011, and who had their weight recorded at the first antenatal visit, were identified from the AMND databank. The women were grouped according to their BMI, calculated from height and weight measurements taken at the first antenatal visit, according to WHO criteria: underweight (BMI <18.5 kg/m^2^), normal weight (BMI 18.5–24.9 kg/m^2^), overweight (BMI 25–29.9 kg/m^2^) and obese (BMI ≥30 kg/m^2^). Relevant details about the index pregnancy were retrieved from the AMND, including maternal age, parity, area-based socioeconomic status, prior self-reported history of diabetes, history of hypertension, gestation at which weight was measured, gestation at delivery, and offspring birthweight, sex and date of birth, from which age at linkage was calculated.

Offspring birth records were linked to a 2011 extract of SCI-Diabetes using the Community Health Index number, a unique individual identifier used on health records in Scotland, or, if this was unavailable, by probabilistic matching on surname, date of birth and postcode (zipcode), to identify individuals with a diagnosis of diabetes.

The SCI-Diabetes database was established at a national level in 2000 and contains demographic and clinical data relevant to diabetes care. It is populated by daily downloads from primary and secondary care databases across Scotland, with increasing completeness since 2004. A validation study among the subset of people with diabetes mentioned on a hospital record in 2007 found that 99% were included in the diabetes register [15]. In SCI-Diabetes, type of diabetes (type 1, type 2, etc.) is recorded by clinicians using a drop-down menu in the electronic health records. To improve the accuracy of this variable for research purposes, an algorithm which combines information from the clinician-recorded diabetes type variable and prescription data is used [16, 17]. Thus, in the current dataset the date of diabetes diagnoses in the offspring ranged from 1956 to 2011, with diagnoses available from birth for all those born after 2000.

Ethics approval for the creation and analysis of the linked dataset containing no personal identifying information was obtained from the London–Dulwich Research Ethics Committee (reference 16/LO/1410). Approvals were also obtained from the steering committee of the AMND, the Scottish Diabetes Research Network epidemiology subgroup committee and the privacy advisory committee of Information Services Division (ISD) Scotland. The pseudonymised dataset was stored and analysed in the Grampian Data Safe Haven.

### Statistical analysis

Data were analysed using IBM SPSS Statistics version 24 (IBM, Armonk, NY, USA). Differences in maternal and offspring characteristics according to BMI category were tested using ANOVA for continuous variables and χ^2^ tests for categorical variables. The effect of maternal obesity on offspring incidence of any diabetes, type 1 diabetes or type 2 diabetes was tested using Cox proportional hazards models, stratifying for year of birth and adjusting for sex of the offspring, comparing outcomes in the offspring of mothers whose pregnancy BMI was underweight, overweight or obese, with those of women with normal BMI. Analyses were adjusted for potential confounding factors, including maternal history of diabetes before pregnancy, maternal history of hypertension, maternal age at delivery, gestation when weight was measured, deprivation category and parity. For the two variables with missing data, on deprivation category and gestation when weight was measured, the missing data were coded to their own categories. In sensitivity analyses we first repeated the analyses after exclusion of women with a history of diabetes prior to pregnancy, to study associations between maternal obesity and offspring outcomes in women without known diabetes. We then repeated the analyses limiting them to those born between 1950 and 1976, i.e. with similar duration of follow-up to those in whom we observed a link between mothers being overweight or obese and offspring cardiovascular events and mortality rates [2]. In addition, we repeated the analyses after excluding women with diabetes prior to pregnancy and with diabetes diagnosed during or after pregnancy. Finally, we examined interactions by sex and repeated the analyses separately for male and female offspring. These interaction models were adjusted for the main effects of sex and maternal BMI.

## Results

Table 1 shows the characteristics of the 118,201 mothers and their offspring. Among the mothers, 24.8% were overweight and 9.5% were obese. The proportion of mothers who were obese increased fivefold between 1950 and 1959 and 2000 and 2011 from 3.1% (*n* = 468) to 15.7% (*n* = 4490) (*p* < 0.0001). Obese women were older and had higher parity and lower deprivation category. The gestation at which weight was first measured was significantly later in overweight and obese women. Among the offspring, 732 (0.6%) had a record of diagnosed diabetes. Among offspring with diabetes, offspring of obese mothers had the highest BMI. More male offspring were diagnosed with diabetes, compared with female offspring, across all maternal BMI categories.

**Table 1:** Characteristics of 118,201 mothers and their offspring according to maternal BMI

Characteristic^a^	Underweight *n*=5283	Normal weight *n*=72,329	Overweight *n*=29,325	Obese *n*=11,264	*p* value
Mothers					
Age, years	24.8 (5.2)	26.1 (5.5)	27.1 (5.6)	27.7 (5.6)	<0.001
Primiparous, *n* (%)	4146 (78.5)	54,611 (75.5)	20,807 (71.0)	7963 (70.7)	<0.001
Deprivation category^b^					
1–2, *n* (%)	1708 (32.3)	22,357 (30.9)	7057 (24.0)	1690 (15.0)	<0.001
3–4, *n* (%)	1481 (28.0)	23,663 (32.7)	10,783 (36.8)	4427 (39.3)	
5–6, *n* (%)	1391 (26.3)	15,477 (21.4)	7000 (23.9)	1784 (15.8)	
Gestational weight measured, weeks	12.23 (4.2)	14.22 (6.7)	16.21 (8.6)	15.64 (9.2)	<0.001
History of diabetes before pregnancy, *n* (%)	3 (0.06)	148 (0.2)	157 (0.5)	77 (0.7)	<0.001
History of hypertension, *n* (%)	117 (2.2)	1904 (2.6)	924 (3.2)	383 (3.4)	<0.001
Any diabetes, *n* (%)	152 (2.9)	2033 (2.8)	1284 (4.4)	860 (7.6)	<0.001
Offspring					
Any diabetes					
Total, *n* (%)	31 (0.59)	437 (0.60)	187 (0.64)	77 (0.68)	0.735
Age at diagnosis, years	32.1 (21.1)	28.5 (19.1)	27.0 (18.3)	26.2 (17.2)	0.393
BMI, kg/m^2^	27.1 (6.8)	28.4 (7.2)	29.7 (7.5)	32.2 (9.2)	<0.001
Male, *n* (%)	24 (77.4)	309 (70.7)	118 (63.1)	55 (71.4)	0.181
Type 1 diabetes					
Total, *n* (%)	14 (0.27)	242 (0.33)	112 (0.38)	42 (0.37)	0.458
Age at diagnosis, years	11.1 (8.5)	14.3 (10.4)	14.5 (9.6)	13.3 (8.6)	0.621
BMI, kg/m^2^	22.2 (3.8)	24.7 (5.3)	25.6 (4.5)	26.9 (5.8)	0.015
Male, *n* (%)	10 (71.4)	155 (64.0)	68 (60.7)	27 (64.3)	0.851
Type 2 diabetes					
Total, *n* (%)	17 (0.32)	193 (0.27)	74 (0.25)	35 (0.31)	0.664
Age at diagnosis, years	49.3 (8.7)	46.2 (11.0)	45.4 (10.8)	41.7 (11.0)	0.068
BMI, kg/m^2^	30.9 (6.2)	32.7 (6.7)	34.9 (7.5)	37.6 (8.9)	0.001
Male, *n* (%)	14 (82.4)	153 (79.3)	49 (66.2)	28 (80.0)	0.125

Data are mean (SD), except where indicated as *n* (%)

^a^Complete data available for all variables other than deprivation category and gestation weight measured where data were available for 100,397 and 112,340 women, respectively

^b^Deprivation category according to area socioeconomic status: 1–2, most deprived; 3–4, middle; 5–6, least deprived

### Maternal obesity and any diabetes in the offspring

Table 2 shows the HRs and 95% CIs for any diabetes in offspring according to maternal BMI category. There was a significant increase in HR of any diabetes in the offspring of mothers who were overweight or obese. The associations were independent of all assessed covariates: after adjustment for gestation when weight was measured, maternal age at delivery, parity, deprivation category, maternal history of diabetes before pregnancy and sex of the offspring, mother being overweight or obese was still associated with over 1.3- and 1.8-fold increased HR, respectively, of any diabetes in the offspring.

**Table 2:** Offspring diabetes according to maternal BMI

Offspring diabetes	HR	95% CI	*p* value	Adjusted HR	95% CI	*p* value
Any diabetes						
Underweight	0.96	0.67, 1.38	0.829	0.94	0.65, 1.36	0.739
Normal weight	1			1		
Overweight	1.23	1.03, 1.46	0.020	1.26	1.06, 1.51	0.009
Obese	1.78	1.38, 2.26	<0.001	1.83	1.42, 2.35	<0.001
Type 1 diabetes						
Underweight	0.88	0.51, 1.51	0.639	0.88	0.51, 1.50	0.628
Normal weight	1			1		
Overweight	1.14	0.91, 1.42	0.27	1.16	0.92, 1.46	0.204
Obese	1.23	0.88, 1.70	0.23	1.25	0.89, 1.75	0.192
Type 2 diabetes						
Underweight	1.07	0.65, 1.76	0.78	1.05	0.64, 1.74	0.835
Normal weight	1			1		
Overweight	1.34	1.03, 1.75	0.032	1.39	1.06, 1.83	0.018
Obese	3.33	2.31, 4.78	<0.001	3.48	2.33, 5.06	<0.001

Adjusted HR, analyses were adjusted for maternal history of diabetes before pregnancy, maternal history of hypertension, maternal age at delivery, gestation when weight was measured, deprivation category, parity and sex of offspring

#### Maternal obesity and type 1 diabetes in offspring

Overall, 410 (0.3%) of the offspring had a diagnosis of type 1 diabetes. Age at diagnosis of type 1 diabetes ranged from 1 to 52 years. Table 2 shows the HRs and 95% CIs for type 1 diabetes in the offspring according to maternal BMI category. In unadjusted analyses and analyses adjusted for potential confounding factors, there was no association between mothers being overweight or obese and HR of type 1 diabetes in the offspring.

#### Maternal obesity and type 2 diabetes in offspring

**Fig. 2:**
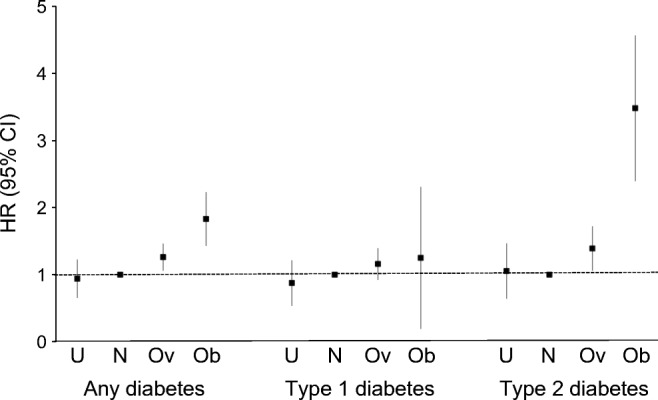
Offspring diabetes according to maternal BMI. U, underweight; N, normal weight; Ov, overweight; Ob, obese

Among the offspring, 319 (0.3%) had a diagnosis of type 2 diabetes. Age at diagnosis of type 2 diabetes ranged from 10 to 61 years. Table 2 and Fig. 2 show the HRs and 95% CIs for type 2 diabetes in the offspring according to maternal BMI category. There was a significant increase in the HR of type 2 diabetes in the offspring of mothers who were overweight or obese. The associations were independent of all assessed covariates: after adjustment for covariates, mothers being overweight or obese was still associated with over 1.4- and 3.5-fold increased HRs, respectively, of type 2 diabetes in the offspring.

#### Sensitivity analyses

Associations between maternal obesity and any diabetes and type 2 diabetes in the offspring remained significant when the 385 women with a history of diabetes prior to pregnancy were excluded from the analyses (electronic supplementary material [ESM] Table 1). These associations also remained significant after excluding all women with diabetes before, during or after pregnancy (ESM Table 1). When restricting the analyses to offspring born between 1950 and 1976, maternal obesity was associated with a 2.3-fold increased HR of offspring type 1 diabetes, as well as a 3.7-fold increased HR of offspring type 2 diabetes and a 3.3-fold HR of any diabetes (ESM Table 2). In individuals born after 1977, maternal obesity was associated with a 2.6-fold increased HR of type 2 diabetes.

#### Sex differences in outcomes

While the overall interactions between maternal BMI and sex of the offspring in predicting offspring HR of any diabetes and type 2 diabetes were marginal (*p* = 0.084 and *p* = 0.076), there were significant interactions specifically for maternal overweight vs normal weight and sex of the offspring in predicting any diabetes (*p* = 0.029 for interaction) and type 2 diabetes (*p* = 0.015 for interaction) in the offspring. Offspring any diabetes and type 2 diabetes were more strongly associated with mothers being overweight in female than in male offspring. The HRs and 95% CIs for any diabetes and type 2 diabetes in offspring born to overweight mothers were 1.50 (1.12, 2.01) and 2.26 (1.37, 3.72) for female offspring and 1.11 (0.89, 1.37) and 1.11 (0.81, 1.53) for male offspring, respectively. There were no interactions by sex on offspring risk of type 1 diabetes (*p* ≥ 0.407).

## Discussion

In this large cohort study including over 60 years of pregnancy data linked to the national diabetes dataset we found that, for pregnant women, being overweight or obese was associated with an increased incidence of type 2 diabetes in the offspring. This association with type 2 diabetes may lie on the causal pathway to explain the link between being overweight or obese during pregnancy and offspring cardiovascular disease and mortality rates [2, 3], and it highlights a potential target for intervention.

Our finding of a link between being overweight or obese and type 2 diabetes in the offspring is in accord with previous observations of associations of pregnant women being overweight and higher maternal weight with increased incidence of type 2 diabetes in the offspring [11, 12], and of maternal obesity with markers in the offspring of increased insulin resistance in childhood [18] and young adulthood [5, 11, 19]; it is also consistent with the findings of increased diabetes risk in offspring born to overweight mothers in the Helsinki Birth Cohort Study [3]. Although the latter study could not distinguish between types of diabetes, the association between being overweight and risk of diabetes was stronger in women than in men, as observed in our dataset. The reasons for a sex difference in the findings are unknown but are possibly related to the smaller pancreatic volume [20], and hence beta cell reserve, in women compared with men, potentially programmed in utero [21], and the findings are consistent with those of other studies suggesting women may be more vulnerable to adverse metabolic programming influences [22, 23]. Evidence suggests increasing incidence of type 1 diabetes in children born between 1989 and 2003 [24], a time when rates of maternal obesity were dramatically rising, as also observed in our dataset. Associations between maternal obesity and childhood-onset type 1 diabetes have been reported in some studies [6–8] but not others [9, 10]. Notably, two studies from Sweden observed an increased incidence of childhood type 1 diabetes in obese mothers without diabetes [6, 8]. We were unable to replicate this finding in our dataset. Reasons for the differences between the Swedish and Aberdeen cohorts are unknown and we only found an association of maternal obesity with offspring type 1 diabetes when we restricted the cohort to individuals who had at least 35 years of follow-up.

The underlying mechanisms linking mothers being overweight/obese to type 2 diabetes in the offspring are not known. The fetal overnutrition hypothesis suggests that the adverse in utero environment associated with maternal obesity, including high circulating levels of glucose, insulin and NEFA, programmes adverse offspring outcomes [25]. In addition, obesity in pregnancy is associated with complex neuroendocrine, metabolic and immune/inflammatory changes which likely impact on fetal hormonal exposure and nutrient supply [26, 27]. It is also thought that epigenetic factors in the intrauterine environment of obese mothers may initiate beta cell stress, metabolic dysregulation and earlier onset of type 2 diabetes and cardiometabolic risk [28]. Further, although we adjusted for both prenatal and postnatal environmental influences in our statistical analyses, it is extremely likely that there will be residual confounding by environmental factors that we were unable to measure. The Hyperglycaemia and Adverse Pregnancy Outcome (HAPO) study showed an association between increased maternal BMI and fetal hyperinsulinaemia, independently of maternal glycaemia [29], supporting an intrauterine influence of maternal obesity on glucose metabolism in the offspring. Others reported that rapid growth in the postnatal environment from birth onwards, rather than during fetal life, was associated with higher insulin levels in childhood [30]. Further studies are needed to understand the contribution of pre- and postnatal mechanisms linking maternal obesity with type 2 diabetes in the offspring.

The strengths of our study include the large sample size and the detailed quality of the antenatal records, including maternal history of type 1 diabetes. Our findings remained similar if we adjusted for maternal diabetes prior to pregnancy in the analyses or excluded women with a history of diabetes both prior to and after pregnancy from the analyses. In addition, we used national population-based health data for diagnosed diabetes (SCI-Diabetes), which allowed us to examine outcomes of both type 2 and type 1 diabetes in the same dataset. A further major strength of our study is that we included outcome data for offspring from birth to age 62 years, which allowed us to explore outcomes across the lifespan. Scottish Diabetes Survey data indicate that type 2 diabetes prevalence increases with age to about 11% for 55- to 59-year-olds (i.e. the oldest age group in this study) [13]. To our knowledge, there are no other databases worldwide that would allow such linkage between maternal obesity records in pregnancy and confirmed diagnosis of diabetes in the offspring. The main limitation is that as only maternal history of type 1 diabetes is recorded in the AMND, we were not able to use ‘gestational diabetes’ or ‘type 2 diabetes’ as covariates to determine whether there was a difference in offspring outcomes of mothers who were obese but did not have diabetes during pregnancy or who were both obese and had gestational or type 2 diabetes. However, we believe the number of women with pre-existing type 2 diabetes in this dataset would be negligible, particularly for mothers who were pregnant in the 1950s and 1960s. In addition, the definition of gestational diabetes has changed over the study period and more detailed measurements of glucose levels taken during pregnancy would be required to generate a comparable definition. As hypertension is part of the metabolic syndrome, we included history of hypertension in our models as a proxy for insulin resistance. Data on maternal gestational weight gain were not available, although a recent study found no evidence that gestational weight gain influenced risk of childhood type 1 diabetes [8]. A further limitation of our study is that we did not have data for all offspring on BMI or lifestyle factors (e.g. diet, exercise) that are known to increase the risk of type 2 diabetes. Indeed, it is plausible that our observations were due to increased BMI in the child, either through prenatal programming of BMI or through shared lifestyle of mother and child. In either case, our observations are important, as pregnancy represents a potential time to intervene with health advice for the family.

In conclusion, we showed that, in pregnant women, being overweight or obese was associated with increased type 2 diabetes incidence in the offspring, independently of perinatal and sociodemographic covariates and maternal history of diabetes. With the rising prevalence of being overweight/obese in women of childbearing age (for example, recent data indicated that over 60% of women in the USA were overweight or obese at the time of conception [4]), our findings have profound public health implications. There is an urgent need to establish effective approaches to prevention of obesity and diabetes among mothers and their offspring.

## Electronic supplementary material

ESM (PDF 80 kb)

## Data Availability

Data are stored in the Grampian Data Safe Haven and are available on request.

## Abbreviations

AMND: Aberdeen Maternity and Neonatal Databank
ISD: Information Services Division
SCI-Diabetes: Scottish Care Information–Diabetes
