# Supplementary material for: Consequences of being overweight or obese during pregnancy on diabetes in the offspring: a record linkage study in Aberdeen, Scotland
Source: Diabetologia. 2019 Jun 19;62(8):1412–9. doi: 10.1007/s00125-019-4891-4 (PMC6647186; doi:10.1007/s00125-019-4891-4)
Supplement: Supplementary file 1 — (PDF 80 kb) [file 125_2019_4891_MOESM1_ESM.pdf]

Electronic Supplementary Material Lahti-Pulkkinen et al

ESM Table 1 Offspring diabetes according to maternal BMI after exclusion of (i) n=385 women with history of diabetes prior to pregnancy and (ii) n=4329 women with history of diabetes before or after pregnancy

	(i) Exclusion of n=385 women with history of diabetes prior to pregnancy							(ii) Exclusion of n=4329 women with history of diabetes before or after pregnancy		
	**HR**	**95% CI**	**p-value**	**Adj HR***	**95%CI**	**p-value**		**Adj HR***	**95%CI**	**p-value**
Any diabetes										
**Underweight**	0.97	0.67, 1.40	0.899	0.97	0.67, 1.39	0.854		0.85	0.56, 1.28	0.434
**Normal weight**	1			1				1		
**Overweight**	1.23	1.04, 1.47	0.018	1.23	1.03, 1.46	0.020		1.26	1.05, 1.52	0.015
**Obese**	1.78	1.39, 2.28	<0.001	2.11	1.80, 2.35	<0.001		1.67	1.26, 2.22	<0.001
Type 1 diabetes										
**Underweight**	0.90	0.52, 1.54	0.690	0.91	0.53, 1.56	0.719		0.92	0.54, 1.59	0.774
**Normal weight**	1			1				1		
**Overweight**	1.14	0.91, 1.44	0.246	1.14	0.91, 1.43	0.263		1.17	0.93, 1.49	0.187
**Obese**	1.23	0.88, 1.72	0.230	1.20	0.86, 1.69	0.278		1.26	0.88, 1.81	0.209
Type 2 diabetes										
**Underweight**	1.07	0.65, 1.76	0.785	1.07	0.65, 1.76	0.785		0.82	0.43, 1.59	0.537
**Normal weight**	1			1				1		
**Overweight**	1.34	1.03, 1.76	0.032	1.33	1.02, 1.75	0.037		1.38	1.01, 1.88	0.040
**Obese**	3.33	2.31, 4.78	<0.001	3.27	2.26, 4,74	<0.001		2.96	1.88, 4.66	<0.001

*adj HR (adjusted hazard ratios). Analyses were stratified for year of birth and adjusted for, maternal history of hypertension, maternal age at delivery, gestation when weight was measured, deprivation category, parity and offspring sex.

ESM Table 2. Post hoc analysis suggested by a reviewer.

Offspring diabetes according to maternal BMI including only individuals born 1950-1976 (n=31371)						
	**HR**	**95% CI**	**p-value**	**Adj HR***	**95%CI**	**p-value**
Any diabetes						
**Underweight**	1.04	0.66, 1.64	0.829	1.03	0.65, 1.63	0.903
**Normal weight**	1			1		
**Overweight**	1.22	0.95, 1.58	0.020	1.27	0.98, 1.65	0.074
**Obese**	3.16	2.22, 4.51	<0.001	3.34	2.31, 4.83	<0.001
Type 1 diabetes						
**Underweight**	0.92	0.33, 2.54	0.876	0.94	0.34, 2.61	0.905
**Normal weight**	1			1		
**Overweight**	0.73	0.39, 1.36	0.320	0.73	0.39, 1.38	0.332
**Obese**	2.35	1.07, 5.17	0.033	2.33	1.03, 5.35	0.039
Type 2 diabetes						
**Underweight**	1.09	0.65, 1.82	0.747	1.07	0.64, 1.79	0.747
**Normal weight**	1			1		
**Overweight**	1.39	1.04, 1.85	0.025	1.41	1.08, 1.94	0.025
**Obese**	3.50	2.35, 5.22	<0.001	3.74	2.48, 5.66	<0.001
**Offspring diabetes according to maternal BMI including only individuals born 1977-2011 (n=86830)**						
	**HR**	**95% CI**	**p-value**	**Adj HR***	**95%CI**	**p-value**
Any diabetes						
**Underweight**	0.88	0.48, 1.61	0.670	0.86	0.47, 1.59	0.636
**Normal weight**	1			1		
**Overweight**	1.19	0.95, 1.51	0.132	1.24	0.98, 1.57	0.076
**Obese**	1.23	0.88, 1.72	0.222	1.28	0.91, 1.80	0.151
Type 1 diabetes						
**Underweight**	0.87	0.46, 1.65	0.671	0.87	0.46, 1.64	0.660
**Normal weight**	1			1		
**Overweight**	1.21	0.95, 1.55	0.122	1.26	0.98, 1.61	0.070
**Obese**	1.11	0.77, 1.60	0.563	1.16	0.91, 1.68	0.417
Type 2 diabetes						
**Underweight**	0.93	0.12, 6.92	0.942	0.84	0.11, 6.31	0.868
**Normal weight**	1			1		
**Overweight**	1.04	0.47, 2.29	0.922	1.06	0.48, 2.35	0.885
**Obese**	2.59	1.09, 6.13	0.031	2.59	1.08, 6.24	0.033

*adj HR (adjusted hazard ratios). Analyses were stratified for year of birth and adjusted for, maternal history of hypertension, maternal age at delivery, gestation when weight was measured, deprivation category, parity and offspring sex.
